# Energy Communities and the Tensions Between Neoliberalism and Communitarianism

**DOI:** 10.1007/s11948-021-00359-w

**Published:** 2022-01-05

**Authors:** Erik Laes, Gunter Bombaerts

**Affiliations:** 1 IE&IS School of Innovation Sciences, Philosophy and Ethics Research Group, Eindhoven University of Technology, De Rondom 70, 5612 AP Eindhoven, The Netherlands; 2 VITO Transition Platform, VITO Vision On Technology, Boeretang 200, 2400 Mol, Belgium

**Keywords:** Michel Foucault, Energy community, Neoliberalism, Governmentality, Communitarianism, Renewable energy directive

## Abstract

The convergent development of (renewable) distributed electricity sources, storage technologies (e.g., batteries), ‘big data’ devices (e.g., sensors, smart meters), and novel ICT infrastructure matching energy supply and demand (smart grids) enables new local and collective forms of energy consumption and production. This socio-technical evolution has been accompanied by the development of citizen energy communities that have been supported by EU energy governance and directives, adopting a political narrative of placing the citizen central in the ongoing energy transition. But to what extent are the ideals that motivate the energy community movement compatible with those of neoliberalism that have guided EU energy policy for the last four decades? Using a framework inspired by Michel Foucault’s idea of governmentality, we analyze the two political forms from three dimensions: ontological, economic and power politics. For the ontological and the economic dimensions, neoliberal governmentality is flexible enough to accommodate the tensions raised by the communitarians. In the dimension of power politics however, the communitarian logic does raise a fundamental challenge to neoliberal governmentality in the sense that it explicitly aims for a redefinition of the ‘common good’ of society’s energy supply based on democratic premises.

## Introduction

In recent years, the European Union (EU) has reconfirmed its objective of becoming a climate neutral economy by 2050, in line with the Paris agreement to keep global warming within 1.5–2.0 °C above pre-industrial levels.^1^ Especially for EU countries not relying on nuclear power, this implies that renewable energy will have to provide the bulk of their energy provision in 2050. The ongoing EU energy transition is not only making the shift from fossil (and in some countries nuclear) to renewable energy sources, but it also rethinks how energy is produced and distributed. Next to traditional top-down distribution, with electricity being generated in large power plants and transported to the end consumer, local energy networks gain in importance. Decentralized renewable electricity (RE) sources have been on the rise for more than a decade, but now the combination with the emergence of storage technologies (e.g., batteries), ‘big data’ devices (e.g., sensors, smart meters), and novel ICT infrastructure matching energy supply and demand (smart grids), enables new local and collective forms of energy consumption and production.

In Recital 70 of the recast Renewable Energy Directive (EU 2019/2001) (RED II), EU officials point out that the participation of citizens in local energy projects has resulted in substantial added value in terms of local acceptance, access to additional private capital for local investment and more choice for consumers.^2^ The “Clean Energy for All Europeans” legislative package (in short, clean energy package), composed of eight separate pieces of legislation (including REDII), emphasizes the need for more bottom-up initiatives and ‘energy democracy’ to further harness the potential of local energy communities (European Commission, 2019). This enthusiastic embrace of community energy in the official EU discourse and legislative framework seems odd due to the fundamental difference between the reality of the present EU energy market – formed by four decades of neoliberal EU energy policy (cf. “Neoliberal EU energy governmentality”) resulting in a mainly centralized electricity production infrastructure owned and managed by a handful of energy multinationals (Engie, EDF, Eon, Vattenfall, etc.) – and the vision and practices advocated by the energy community movement following a communitarian logic – i.e., one of bottom-up democracy, citizen ownership and independence from traditional energy companies.^3^ While the tendency in the literature is to view the adoption of such socio-technical innovations in dualistic terms – i.e., they either contribute to a radical system transformation or to system reproduction (Wittmayer et al., 2021) – these extremes should be seen as the outer limits of a set of possible future energy system configurations involving a multitude of different interconnections between energy communities and the incumbent energy system. Since the official embrace of community energy in EU energy policy is only in its nascent stage, our aim in this paper is not to predict the outcomes of this new policy package, but rather to analyze the adoption of energy communities in terms of the representative characteristics (and in some instances, opposing poles) of two underlying government logics (or, in the words of the French philosopher Michel Foucault, ‘governmentalities’): neoliberalism and communitarianism.

By coining the concept of governmentality, Foucault (2007) wanted to describe a new form and logic of state policy, which opposes the sovereign exercise of power. Whereas the problem of the sovereign exercise of power concerns primarily the maintenance of the transcendental relationship that the sovereign has with his territory (and only secondarily with the subjects inhabiting this territory), modern states from the sixteenth century onwards were increasingly confronted with the disappearance of feudal connections and the self-evidence of the ‘Divine Law’ from which sovereignty derives its legitimacy. The new form of governmental state policy can therefore only manifest itself immanently – i.e., in the field of existing social relations – and is thereby constituted as an extension of other forms of governance (e.g., the pater familias as head of the family, the abbot as head of a convent, the production manager as head of the factory). Governmentality should therefore be understood as a set of techniques, procedures, institutions and calculations with which an authoritative institution tries to steer the conduct of individuals or collectives in the social field. In more concise terms, governmentality can be defined as ‘the conduct of conduct’ (Foucault, 1982, p. 220).

By analyzing the adoption of energy communities in EU policy in terms of the underlying logics of neoliberalism and/or communitarianism, we aim to explain the alignments and points of friction between both forms of governmentality. We thereby show where a space for possible compromises exists, but also where such compromises might be difficult to reach and hence a choice in favor of one type of governmentality imposes itself. The wide variety in the way the notion of neoliberalism is used (Ferguson, 2010) forces us first to provide some clarification on this key term that animates our discussion. Therefore, we first demonstrate how the Foucauldian conception of neoliberalism – and the distinction between neoliberalism understood as an ideology or doctrine, and as an ‘art of government’ – serves us well to draw out important characteristics of EU energy governance in three dimensions: ontological politics, economic politics and power politics. The next section (“Foucault’s conception of neoliberal governmentality”) first introduces the Foucauldian take on neoliberalism, while the section “Neoliberal EU energy governmentality” discusses the dominant characteristics of EU energy policy when analyzed through a Foucauldian lens. We then proceed with asking ourselves the question whether, and on what account, the form of governmentality propagated by (part of) the energy community movement is compatible or at odds with neoliberal governmentality.

In the section on “Communitarian energy governmentality” we therefore discuss this form of governmentality along the three dimensions mentioned earlier. We conclude that for two of these dimensions (the ontological and the economic dimension), practical compromises between neoliberalism and communitarianism are very well conceivable. In the dimension of power politics however, the communitarian logic does raise a fundamental challenge to neoliberal governmentality in the sense that it explicitly aims for a redefinition of (the control over) the common good of society’s energy supply based on democratic premises. In line with the overall Foucauldian gist of our argument, we refrain from expressing a value judgement on this state of affairs, but that does not mean that there are no dangers involved for the energy community movement as they are drawn into playing along the rules of the neoliberal ‘game’. In the “Discussion” section, we discuss some of the dangers of ‘betraying’ the communitarian political agenda in relation to practices of localization, volunteering and internal democracy. We conclude with an overview of our argumentation and underline the need for a research agenda on energy communities that moves beyond abstract ethical discussions. Instead, future research should look at the concrete and practical implementations and realizations of energy communities in the field, in order to understand the opportunities, tensions and dangers opened by embracing hybridizations of neoliberalism and communitarianism in the energy system.

## Foucault’s Conception of Neoliberal Governmentality

In today’s discussions, the most common usage of ‘neoliberalism’ in political philosophy refers to a doctrine based on the premises of privatization (i.e., the expropriation of common goods), valorizing private enterprise, creating markets, and molding the state apparatus based on a private enterprise model (Harvey, 2007). On this account, neoliberalism should be seen as a ‘utopia’ (in the literal sense that a fully neoliberal state can never be realized), which nevertheless as a doctrine inspires the general direction of policy design (Ferguson, 2010). In his lectures on *The Birth of Biopolitics*, Foucault (2008) develops the view that neoliberalism should be defined and studied as a form of governmentality or as a ‘political technology’, i.e., as a coherent set of practices and techniques to ‘conduct the conduct’ of people, and thus not as a doctrine or ideology.

Taking his cue from the German neoliberalist school, Foucault argues that neoliberalism does not have any essential characteristics or a fixed form (Lagasnerie, 2012; Lemke, 2001).^4^ Neoliberalism does not reduce the essential nature of human beings to the nature of a ‘homo oeconomicus’, but it approaches human beings in certain aspects of their behavior as ‘homines oeconomici’. It seduces human beings (or organizations) to adopt entrepreneurial behavior by relying on carefully designed, artificially induced and skillfully monitored conditions (Oksala, 2016). (Neo)liberalism does however have a kind of hard core – what Foucault calls “une armature originale” – or a recurring management style. Neoliberals want to limit state power. This art of government revolves around imposing an internal limit to state governance; more precisely yet it imposes a self-limitation. While classical liberalism mainly imposes limits on state intervention in order to avoid disturbing processes of commercial self-organization and market formation (i.e., in the form of traditional economic “*laissez-faire*” policy), neoliberal thought stresses the need for governments to be active in stimulating the promulgation of markets, also beyond strictly commercial domains, since “*…*pure competition is not a primitive given. It can only be the result of lengthy efforts and, in truth, pure competition is never attained. Pure competition is and can only be an objective, an objective thus presupposing an indefinitely active policy. Competition is therefore the historical objective of governmental art and not a natural given that must be respected” (Foucault, 2008, p. 120). Instead, government should enact what Foucault calls ‘conformable actions. The goal of these actions is not to intervene on the mechanisms of the market economy, but on the conditions of the market (Foucault, 2008, 138). Government action on this level establishes what the German neoliberals call the ‘framework’ of the market – i.e., the set of applicable laws, access to technology, the level of education of the population, etc.

Summarizing, for Foucault neoliberalism is first and foremost a praxis – i.e., a practical way of governing the conduct of people and organizations – permanently accompanied by a reflection on the strategies, tactics, target domains and goals of practical governance interventions in terms of their impact on market mechanisms. As for other practically oriented technologies, the questions whether the technology works and what its effects are remain for neoliberal governmentality the most important ones. This also implies that monitoring, learning, and adapting to changing circumstances should be vital ingredients of the approach. To proceed with our argument, we first explain how and why the EU’s clean energy package should be seen as a form of neoliberal governance as outlined by Foucault.

## Neoliberal EU Energy Governmentality

Following earlier neoliberal reforms in the United States and the United Kingdom, the European Commission (EC) began liberalizing the EU electricity and gas markets in the 1990s through a series of directives aimed at creating a single European energy market (Glachant, 2003). Eliminating vertical organization by targeting in particular the unbundling of public services in the electricity sector (such as the German ‘Stadtwerke’ managed by local authorities), and the privatization of state-owned energy companies were the key ingredients of this strategy (Verbruggen et al., 2015). In addition to an interwoven with this neoliberal agenda, the EU is also actively pursuing a decarbonization agenda. Market-based instruments such as the carbon emission permit trading scheme also claim center stage in this agenda for already more than a decade (Verbruggen et al., 2019). The clean energy package is perhaps the most ambitious program worldwide to respond to climate change, which at the same time further pursues the agenda of strengthening the internal EU energy markets. Whilst a detailed analysis of the entire package is beyond the scope of this article, this section will analyze some of its key dimensions in relation to the Foucauldian outline of neoliberalism, with a particular focus on the role of energy communities. In turn we discuss the ‘way of being’ instigated by the package (“Ontological politics”), the economic instruments adopted (“Economic politics”), and its discourse on the power of market actors (“Power politics”).

### Ontological Politics

As explained in the previous section, the ‘natural way of being’ from a neoliberal point of view is ‘being an entrepreneur’. There are several ways in which the clean energy package expands entrepreneurship, the most significant of which is through consumers, who are “at the heart of the energy transition” (European Commission, 2019, p. 12). These consumers no longer just buy their energy, but instead are supposed to become active in a whole range of energy practices such as decentralized electricity production (thereby becoming ‘prosumers’) or providing flexibility to the electricity market by relying on smart meters, appliances and grids (European Commission, 2019, p. 13). To this end, the clean energy package secures a new set of citizen rights to generate, self-consume, store, and sell renewable energy as well as participating in energy communities. These new rights are expected to produce a large expansion in community energy entrepreneurship to the point that “by 2030, energy communities could own some 17% of installed wind capacity and 21% of solar. By 2050, almost half of EU households are expected be producing renewable energy” (European Commission, 2019, p. 13). Experimental smart grid project all over the EU provide an indication of how this energy community entrepreneurship could work out in the future. Many of these projects are exploring the idea of coupling electricity prices to grid states, meaning that peaks of electricity availability (e.g., from solar panels at noon) automatically decrease energy prizes just-in-time. A smart grid algorithm then sets the household appliances, heat pumps etc. in motion in accordance with price signals, unless indicated otherwise by the resident/end user. In this way, the smart grid technology nudges households into picking the more economical option without them necessarily having to change their consumption behavior at all or actively deciding to do so. Such ‘smart’ energy communities are built upon the idea that most individuals are driven by personal financial benefits and with this behavior automatically help the community energy grid to operate smoothly. By making the smart grid as convenient as possible for end users, the transition from fossil energy sources to renewables ‘in the background’ will become nearly invisible to them. Hence, a sense of ‘community’ functions only as a kind of latent variable behind an otherwise almost entirely automatically driven market integration of locally produced renewable electricity.

### Economic Politics

Setting up frameworks to create market conditions and found new markets is one of the central roles of the clean energy package. This can be seen on several levels. The goals of these new market boundary conditions include allowing electricity to move more freely by building the necessary network infrastructure, increased flexibility in the sources of electricity, increased market-based investments, and increased ability to respond to electricity market crises (European Commission, 2019). On top of this, we already mentioned the carbon market which reveals a new dimension of market-based governmentality. To this can be added large public investments to get market forces underway, and even greater levels of private investment for a total of “around €180 billion a year” over the next decade (European Commission, 2019, p. 6). Setting up the right market conditions includes the fostering of new market actors such as energy communities (cf. previous section). As a part of the clean energy package, RED II grants renewable energy communities (RECs) specific rights and obligations. EU member states determine the specific legal entities or forms that can be considered RECs (based on e.g., ownership, control, and spatial proximity of participants) while complying with the general definition of RED II. Member states are required, inter alia, to assess the opportunities open to and barriers faced by RECs, and to develop enabling frameworks that allows RECs to access all energy markets on a ‘level playing field,’ neither at advantage nor disadvantage compared to traditional energy companies. These enabling frameworks must include, inter alia, measures to ensure that the participation in the RECs is accessible to all consumers and make sure that tools to facilitate access to finance and information are available.^5^ Moreover, EU member states are required to consider specificities of RECs when designing support schemes in order to allow them to compete for support on an equal footing with other market participants.^6^ The importance of notions such as ‘equal footing’ or ‘level playing field’ makes it very clear that in the clean energy package RECs are mainly positioned as actors that increase competition on the energy market, while simultaneously also delivering social and ecological benefits.

### Power Politics

There is no shortage of critical literature that shows how the EU neoliberal focus on market-based governance has allowed powerful elites to promote public policies that mainly serve to further their interests. Kirkegaard et al. (2021) for instance demonstrate that the new EU competition-based tender processes for RE production capacity serve to strengthen the incumbent multinationals and limit competition by denying the opportunity of smaller actors to take part in the energy system. Verbruggen and Laes (2021) show how the creation of a market for tradeable green certificates (a policy instrument introduced to promote the growth of RE generation) led to excessive financial transfers from small electricity consumers to large-scale renewable electricity generators, limited to no technological innovation, and target fetishism. Nevertheless, both Kirkegaard et al. (2021) and Verbruggen and Laes (2021) also point out that alternative market-based policy designs were available in the cases they discuss. Therefore, the use of market-based policy instruments is not per se inherently problematic, but rather the incumbents’ vastly superior understanding (compared to the often-understaffed policy administrations) of the ‘nuts and bolts’ of these instruments and the lobbying power at their disposal to push through the designs that best fit their interests.

It is therefore certainly a worthwhile critical task to problematize neoliberalism based on the unequal outcomes of the policies that it inspires. However, as pointed out in the above examples, there is often no direct causal link between a neoliberal inspiration and a practical policy design and outcome. Instead, this link is mediated by the power of incumbent players. As pointed out by Foucault, the neoliberal focus on the intensification of competition entails a critique and move away from (quasi-)monopolistic enterprises. In the German version of neoliberalism (ordoliberalism), this also means an expansion of the number of small and individual enterprises or, in the words of the German neoliberal Röpke, “shifting the center of gravity of governmental action downwards” (quoted in Foucault, 2008, p. 148). Some of Röpke’s policy objectives are worth mentioning here because of the obvious connection to the promotion of RECs in the clean energy package, such as the development of what he calls non-proletarian industries (i.e., craft industries and small businesses); decentralization of places of residence, production, and management; and the organic reconstruction of society based on natural communities, families, and neighborhoods (Foucault, 2008, pp. 147–8). Seen from this perspective, the power politics of the clean energy package aim at empowering energy communities on the energy market as a counterweight to the incumbent energy market players, albeit that the meaning of empowerment is clearly restricted to the purely economic sense of increased competition.

## Communitarian Energy Governmentality

The duality between philosophical frameworks or systems based on either individualistic (liberal) or communitarian assumptions or principles goes back to at least Enlightenment debates (e.g., rationalism vs. romanticism). Bell (2020) reports how this debate has been re-ignited mainly by the publication of Rawls’s *A Theory of Justice* (1971) and subsequent criticism from communitarian thinkers such as Alasdair MacIntyre and Charles Taylor. Of relevance to our present discussion is the fact that a recognizable part of the energy community movement embraces an explicitly communitarian logic, mainly expressed in the desire to (re)establish collective ties and to build upon values such as community trust and empowerment, and to establish a culture of ‘do it yourself’ and ‘do it together’ (Seyfang & Haxeltine, 2012). The European Federation of Energy Cooperatives (REScoop.eu) is the most important exponent of this communitarian logic. REScoop.eu tries to actively distance itself from neoliberalism, in particular the British neoliberal policies pursued under Margaret Tatcher, which included an aggressive form of privatization under which “cooperatives were suppressed” (Vansintjan, 2015, p. 27).^7^ But beyond this particular policy of suppression, the vision of neoliberalism to which they oppose themselves remains undefined and indistinct from the crisis tendencies of capitalism and its bubbles (Vansintjan, 2015, pp. 27–9). It is therefore certainly a worthwhile task to investigate how the communitarian governmentality compares to the German neoliberalism as explained by Foucault. In what follows, we specify this communitarian logic further in its three constituent political dimensions: ontological politics, economic politics and power politics.

### Ontological Politics

By arguing that (neo)liberalism starts from the wrong conception of people as ‘atomistic individuals’, communitarians pursue a specific form of ontological politics. Through political argument and action, communitarians want to reinforce the social ties between people, based on the conviction that at a deep ontological level people are inherently social beings. According to the communitarians, denying this sociality of human beings can only lead to feelings of estrangement and alienation, understood as the flipside of having intense engagements with the world or others. This type of argument comes in many forms; here we discuss a relevant example that discusses the role of technology in establishing a community. In his article *Technology as skill and activity*, Coeckelbergh (2012) draws on the insights of Martin Heidegger and Hubert Dreyfus to argue that individuals are always already embodied agents in the world. Far from having the choice of means to realize an autonomously arrived-at conception of the good life (cf. Rawls), these authors advocate that a major part of our daily life is in fact governed by unchosen technically mediated routines and habits. This leads Coeckelbergh to conclude that technologies should be evaluated not only as artefacts with certain impacts on society, but more importantly also in terms of the skills they require from their users and the habits they support. He concludes that technologies that make our engagements with the world and others more intense stand a better chance of making us better persons, while technologies that isolate us from the world and others lead to alienation. Following Foucault’s view on neoliberalism as a political technology, we can extend Coeckelbergh’s criticism to this particular form of governmentality.

What to think of Coeckelbergh’s argument? To answer this question, it is good to remind the reader here that neoliberal governmentality in its ontological politics strives to establish entrepreneurship as the basis of society, and by doing so, imposes an internal limit on the direct exercise of state power. Furthermore, Foucault (2008) even argues that neoliberal governance needs civil society as its counterpart, i.e., as a permanent test of the appropriateness, usefulness or efficiency of its interventions. According to Foucault, civil society should be seen as a ‘transactional entity’ that emerges precisely at the intersection between the ones governing and the ones being governed. Civil society is the entity that enables a spontaneous synthesis of interests out of a previously inchoate bundle of inclinations and desires, that can then serve as the starting point for neoliberal policy interventions to hook unto. Interests are furthermore not limited to egoistic motives: Foucault’s understanding of civil society also leaves room for spontaneous sympathy, for ‘disinterested interests’. And since people’s sympathy usually goes out to concrete communities (e.g., the family, the neighborhood, the village or city, the region), one could even argue that the more fine-grained the organization of civil society becomes (e.g., in local energy communities), the better it will serve its function of testing, challenging and limiting state interventions.

However, through this focus on entrepreneurship, neoliberal governmentality will also not hesitate to engage energy communities into a competition with traditional project developers or companies, for instance to acquire ownership of available spaces to develop RE infrastructures. To the extent that such competition often requires strategizing and quick decision making in the interest of accelerating the energy transition, the needed time for community building around a common project will often be lacking. This represents a significant challenge to communitarian energy governance.

### Economic Politics

Energy communitarians also pursue a particular kind of economic politics, which draws its inspiration mainly from the work of Elinor Ostrom and colleagues on common pool resource management (Ostrom, 2010, 2015). In this work, common pool resources are characterized by two main features: it is difficult to exclude actors from making use of them, while each resource unit consumed by one actor is not available for consumption any more by another actor. Typical cases discussed by Ostrom and colleagues are fisheries, irrigation networks or forestry; but it is clear that public spaces (able to host a wind power farm) or rooftops of public buildings (able to host photovoltaic panels) equally fall under the category of common pool resources. Both on theoretical and empirical grounds, the work of Elinor Ostrom and colleagues has been seminal in showing not only that prisoners’ dilemmas in common pool resource problems can be avoided by forms of collective action, without taking recourse to market (i.e., privatization of the commons), or authoritarian (i.e., central government rule) solutions, but furthermore that these solutions often outperform the market or authoritarian alternatives in terms of efficiency and effectiveness. Crucial to the success of these collective solutions is that participants can communicate on a regular basis with each other on the rules to govern the resource over longer periods of time, fostering relations based on trust. Similarly, in the literature on energy communities, various authors refer to the psychological benefits of having a group of persons who seek shared ends, participate in common activity and experience a sense of togetherness (for an overview, see European Commission Joint Research Centre, 2020). Such communities, based on face-to-face interaction, are furthermore said to be governed by sentiments of altruism in the sense that constituent members have the good of the community in mind and act on behalf of the community’s interest, in terms of generating local value, financial returns, as well as educating and mobilizing citizens (Kunze & Becker, 2014).

So, while there certainly are empirical grounds to support the claim that energy communities contribute to the local common good, the question raises whether the economic politics pursued by the energy community movement puts it at odds with neoliberal governmentality. Two issues stand out here. Firstly, in Ostrom’s model, autonomous individuals with relatively stable preferences (i.e., interests) and bounded rationality (e.g., limited information processing capacities) decide whether to cooperate through cost–benefit calculations striving to maximize their personal welfare. This welfare could include social norms and the welfare of others – i.e., altruism – but this does not take away the fact that Ostrom stresses the personal nature of these cost–benefit considerations. Furthermore, empirical studies indicate that other-regarding attitudes seem to be prevalent mainly in small-scale initiatives; financial motivations do play a role in decisions to join energy communities and become even more prevalent in larger cooperatives (Sloot et al., 2019). The main message here is that none of these communitarian governance arguments conflict with neoliberalism: if community feelings or local benefits stimulate local entrepreneurship, they provide suitable anchor points for neoliberal tactics. Secondly, while the findings of Ostrom and colleagues do invalidate the recourse to neoliberalism as a doctrine (or ideology) – in the sense that privatization of public resources should be seen as a ‘silver bullet’ for the governance of common pool resources – there is nothing in the eight design principles for successful collective action identified in Ostrom (2010) that is in principle irreconcilable with the use of certain neoliberal techniques. For instance, the design principle stating that “appropriation rules are congruent with provision rules; the distribution of costs is proportional to the distribution of benefits” (p. 653) can quite well be managed according to prevalent market principles (e.g., following the commonsense rule that those persons that invest the most in the local resource infrastructure should also benefit the most).

### Power Politics

Finally, a recognizable part of the energy community movement explicitly showcases its political ambitions to regain power and control over the energy system, in terms of redirecting financial investment flows and deciding who benefits from RE production directly (i.e., from the energy flows) and indirectly (i.e., from the profits made by selling energy on the market). As advertised on the website of REScoop.eu (www.rescoop.eu), one of the main arguments used by the cooperative movement is that energy communities are a crucial factor in promoting energy democracy. This claim to energy democracy can be understood in both an internal and external sense, i.e., related to the internal functioning of energy communities, or to the wider impact of the energy community movement on power dynamics in the energy system.

Internally, the claim to democracy is based on Ostrom’s work (cf. “Economic politics”), which has demonstrated the sustainability of governance schemes for common pool resources based on stakeholders acting as equals and building trusted relationships through collective deliberation. Externally, the energy community movement aims for a democratization of the energy transition (Dóci et al., 2015), based on the premise that it acts (or could act) as a counterbalance or even alternative to the power of the traditional incumbent energy companies active on the EU energy markets, who, due to their considerable financial clout, also have the ability to significantly influence national and EU policy making (Haas, 2019). Other authors (Cowell & Devine-Wright, 2018; Slee, 2015; Wirth, 2014) also point out that as an indirect result of participating in an energy community, citizens often also take on other active energy-related roles and thereby help in shaping the energy transition as a whole.

Hence, it is clear that at least part of the energy community movement is striving for a political alternative to neoliberal governmentality, in the sense that it wants to empower citizens directly in taking over control of the financial and energy flows circulating through the energy system. Ironically enough however, as remarked by Deleixhe (2018, p. 71), when taken to its extreme, this political push for communitarian governance could serve exactly the same function of limiting state power as promulgated by neoliberalism: “…the assumption that commons are self-creating, self-regulatory and would function better away from any form of centralized control is so strong that it is sometimes difficult to fathom what distinguishes it from the neoliberal utopia according to which all aspects of societies would be better off being deregulated and abandoned to unimpeded market mechanisms”. Similar to our discussion of neoliberalism as a doctrine (cf. “Foucault’s conception of neoliberal governmentality”), this quote serves as a reference to what we might call the ‘communitarian utopia’. This doctrinal temptation reveals itself most clearly in communitarian discourse advocating a certain critical distance to the traditional institutions of representative democracy. For instance, REScoop.eu’s guidance document on the transposition of the EU Clean Energy Package (REScoop & ClientEarth, 2020) clearly states that effective control of the energy community means that more than 50% of the shares are owned by individual citizens (whereas REDII leaves open the question of effective control and hence does not exclude e.g., municipal governments from controlling RECs).

Thus, while communitarian governmentality directly challenges its neoliberal counterpart on the power dimension, this challenge comes with its own set of particular dangers, directly related to the practice of limiting state power that it shares with neoliberalism. For instance, as is the case for neoliberal governance, proposals for the widespread unchecked adoption of energy communities raise potential fairness issues: as areas with already strong community ties (e.g., rural vs. urban areas) are more likely to develop other community initiatives (such as RECs), they are more likely to become the recipients of scattergun approach state support. Just like the social welfare state implements correction mechanisms to markets (e.g., for fighting energy poverty), specific corrections might be needed also in the case of RECs, especially if this movement grows beyond the level of a niche.

## Discussion

So far, our argumentation has exclusively touched on theoretical and conceptual issues in the debate between neoliberalism and communitarianism: we introduced an understanding of neoliberalism as a form of governmentality (i.e., a coherent set of techniques inspired by, but never fully able to realize the neoliberal utopia) and discussed the ontological, empirical and political arguments mobilized to resist neoliberal interventions. We conclude that for two of these dimensions (the ontological and the economic dimension), neoliberal governmentality is flexible enough to accommodate the challenges raised by the communitarians. In the dimension of power politics however, the communitarian logic does raise a fundamental challenge to neoliberal governmentality in the sense that it explicitly aims for a redefinition of the ‘common good’ of society’s energy supply based on democratic premises.

Following Foucault’s conception of governmentality as a praxis, the ‘truth’ about different forms of governmentality will only be revealed through their inscription in a regime of practices. Applied to our case, this means we need to study practices of forming, implementing and maintaining energy community projects in the field. Though a detailed taxonomy of the many shapes that energy community initiatives can take on is beyond the scope of the present paper – for this we refer the reader to e.g. Moroni et al. (2019) and Sousa et al. (2019) – in this discussion section we merely want to introduce and explore some important tensions that according to us will have to be navigated especially by energy community initiatives that wish to hold true to a communitarian logic in a context of neoliberal EU ‘clean energy’ governmentality. In turn, we discuss these tensions in relation to three different practices that correspond to the three political dimensions discussed in the previous section: practices of localization (addressing the question of how to draw the boundaries of a community, most relevant to the dimension of ontological politics), volunteering (addressing the question of how to account for the role of unpaid labor, most relevant to the dimension of economic politics) and internal democracy (addressing the question of how to organize decision making in energy communities, most relevant to the dimension of power politics).

Firstly, REDII contains a provision that RECs should be effectively controlled by shareholders or members that are located in the proximity of the renewable energy projects that are owned and developed by that legal entity.^8^ However, what the practical implications are of this ‘proximity rule’ is left to the discretion of the EU member states. This provokes a possible tension in the communitarian logic. On the one hand, the realization of large RE projects (such as wind or solar parks) by a large energy cooperative going beyond the border of one municipality contributes effectively to national decarbonization goals, and also adds to the political weight and lobbying power of the cooperative movement as such. On the other hand, the top-down management approach needed to realize such large projects could be perceived as going against the spirit of an energy community, which is more centered around notions of doing good for the local neighborhood or village. A focus on large projects could even potentially turn into a threat to the energy community movement, even though this seems to be an essential ingredient in a strategy of becoming big enough to challenge the energy incumbents on the market. It seems that a careful balance will be needed between the growth imperative and keeping the local character of energy communities intact. This could be achieved for instance by stimulating cooperation between a medium to large (and more professional) cooperative and a local citizens’ initiative, for instance by supporting the local citizens’ initiative through free advice.

Secondly, volunteering is an inherent part of the communitarian culture of ‘do it yourself’ and ‘do it together’ (Seyfang & Haxeltine, 2012). The informality of the energy community logic provides a low barrier entry point for people to get engaged, and thus increases the potential of establishing an energy constituency that is truly inclusive of the entire local environment. However, once up and running, reliance on informal labor is often associated with difficulties in sustaining operations in a highly formalized and competitive energy system. While professionalization holds the prospect of access to resources and enabling the capacity to engage in more complex RE projects, it also comes with a particular set of dangers. Firstly, professionalization might just be ‘a step too far’ for many informal collectives that are not interested in producing energy in the first place, but mainly in engaging with their neighbors and ‘doing good’ for the local environment. Secondly, voluntariness is of crucial importance to energy communities, especially when it comes to local embedding. If the local participants in an energy community also perform paid work, there is a real danger that they will no longer be seen as representatives of the local interest. Nevertheless, given a lack of capacities (e.g., in terms of personnel, skills, leadership, and finance) in most community energy initiatives, professional support will be necessary in most cases. Compromises between the two conflicting demands are however possible. For instance: local initiatives could be professionally supported by energy community network organizations that operate on a higher geographical scale. Other compromises could also be envisaged, e.g., establishing a volunteer board or steering committee for setting the strategic directions of the local initiative, while leaving the implementation to a professional workforce.

Thirdly, energy cooperatives put high value on the democratic nature of their operations. They define themselves as “democratic organizations controlled by their members, who actively participate in setting their policies and making decisions. Men and women serving as elected representatives are accountable to the membership. In primary cooperatives members have equal voting rights (one member, one vote) and cooperatives at other levels are also organized in a democratic manner”.^9^ However, as pointed out by Deleixhe (2018), this claim to democracy functions only under particular circumstances, namely on a strong willingness of all community members to cooperate harmoniously, which seems to be presupposed based on all actors having on a strong sense of belonging to a shared community. For Deleixhe, bottom-up democracy precisely proves its worth in situations where no attempt is (or can be) made to build a totalizing community, and where the constant reality of internal agonistic conflicts is crucial for maintaining a vibrant pluralism.^10^ This theoretical objection is complemented by Van Veelen’s (2018) empirical findings, showing that decision making in community-driven energy communities is in many cases also characterized by inequalities in power, representation and burdens. That these deviations from an ideal democratic functioning do not commonly lead to open debate and controversy can at least partly be explained by the fact that cooperatives often do not engage in projects with high financial risk, and that RE projects in the EU often enjoy guaranteed returns on investment through various mechanisms such as subsidies or feed-in tariffs. To put it bluntly, democratic decision making will likely become a lot easier if every member of the community is guaranteed a stable (but generally limited) return on investment for their share in a RE project.

## Conclusion

With the publication of the EU clean energy package, a window of opportunity for the accelerated growth of energy communities seems to present itself. Following Foucault, we have asked ourselves the question what this ‘tipping point’ in EU policy making reveals about our present situation. By drawing on Foucault’s conception of neoliberalism as a form of governmentality, we were able to show that something much more complex is at hand than a simple opposition of neoliberal and communitarian logics would suggest. In turn, we investigated three political dimensions which at first sight could serve to set apart communitarian governmentality from its neoliberal counterpart: to wit ontological, economic and power politics. We concluded that for two of these dimensions (the ontological and the economic dimension), neoliberal governmentality can be aligned with communitarian challenges. In the power dimension however, the cooperative energy community movement does fundamentally challenge the neoliberal logic, in the sense that they propose a different, more democratic distribution of power in the energy system, based on citizen control of resources. In our view, these findings open a new perspective on the dynamics of the energy community movement in the EU which could be further explored with the help of a Foucauldian toolbox. Here we suggest further investigations along three research lines.

Continuing from the initial explorations we undertook in the “Discussion”, a first line of empirical research focuses on mapping the diversity of energy communities that either arise under the impulse of the clean energy package or grow out of existing initiatives that subsequently become officially labelled as an ‘energy community’ under the stipulations of REDII. The creation, implementation as well as the daily functioning of an energy community rely on certain practices and decisions, which concern e.g., who has access to the community and under what conditions, where the funding of the community comes from, who decides on project investments and based on which rules, how the proceeds of the project are distributed among the members of the community, and so on. For each of these practices, it should be possible to outline how this practice would be shaped from a neoliberal or a communitarian logic; and based on these two ideal types, to determine the space for compromise. In other words, this first research line comes down to a detailed investigation of the various hybrid forms of ‘neoliberal communitarianism’ as they are being realized in the field, by navigating the tensions, oppositions and opportunities revealed by the juxtaposition of neoliberal and communitarian governmentality.

A second line of inquiry concerns the role of national governments in implementing the EU clean energy package. As argued earlier, according to both the neoliberal and the community logic, the state has only a limited role to play. According to both logics, the government should limit itself to formulating the right framework conditions, in the first case to let the market mechanisms do their work, in the second case to allow community initiatives to grow spontaneously. The question then arises as to how exactly the government should shape such framework conditions. For instance: neoliberalism stresses the need for a ‘level playing field’, but in a historical context characterized by major power imbalances between the energy incumbents and community initiatives, the question arises as to how far the government should go to correct these imbalances. Or conversely: for the communitarians, state institutions (including local governments) should have a role only in providing an ‘enabling framework’ for energy community projects but should refrain from taking a leading role in the governance of these communities. Under these conditions, it is an open question how the democratic accountability of the energy community to the local population as a whole could still be ensured.

Finally, a third line of inquiry concerns the power dynamics and politics of the energy community movement. In the section on the “Power politics” of REScoop.eu, we already pointed out that their view on democratic empowerment has both an internal and external democratic component. The default location and scale of action and analysis for internal democracy is taken to be ‘the local’, perceived as both a geographical scale and a set of social relations, for instance in the expressed objectives of localizing ownership and control over energy infrastructures as a priority for achieving greater energy democracy. ‘Local’ and ‘community’ are thereby used as unproblematic categories: it is simply assumed that the desirable social relations for setting up an energy community correlate with the ‘local’ level. From a Foucauldian point of view however, this neglects the power dimension involved in acts of territorialization. The emphasis on the ‘local’ should therefore be analyzed as a potentially contestable act of boundary making, through which the criteria for belonging, and thus the subjects of claims for justice are negotiated in order to determine the allocation of resources. Similarly, on the dimension of external democracy the power politics of REScoop.eu focus particularly on recasting the relationship between the state, the market, and civil society through a reorganization of how and where energy resources are controlled. In pushing forward this agenda throughout the EU, REScoop.eu aims at nothing less than a complete overhaul of energy system governance based on a universally applicable normative model of control. This raises the question of the power dynamics involved in realizing such normative and universalist claims or interpretations of energy democracy, not only in opposition to the incumbent energy players, but also with in competition with other interpretations of energy communities that embed energy democracy in locally and contextually co-constructed practices.

## Footnotes

1 Communication from the Commission to the European Parliament, the European Council, the Council, the European Economic and Social Committee, the Committee of the Regions and the European Investment Bank, *A Clean Planet for all A European strategic long-term vision for a prosperous, modern, competitive and climate neutral economy*, COM/2018/773 final.

2 https://eur-lex.europa.eu/legal-content/EN/TXT/?uri=uriserv:OJ.L_.2018.328.01.0082.01.ENG&toc=OJ:L:2018:328:TOC [accessed on 25 October 2021].

3 See e.g. the reference to the 7 principles of the International Cooperative Alliance on the website of RESCOOP, the EU federation of energy cooperatives (www.rescoop.eu) [accessed on 25 October 2021].

4 Called the ‘Freiburg school’ or the ‘ordoliberals’, after the journal *Ordo*, directed by Walter Eucken, one of the German neoliberal movement’s leading figures. Compared to its American counterpart, as propagated by e.g. Milton Friedman and Friedrich Hayek, ordoliberalism can be described as moderate.

5 Article 22(4)(f) and (g) of the RED II.

6 Article 22(7) of the RED II.

7 REScoop.eu is a network of 1,900 cooperatives that includes over 1.25 million citizens. REScoop.eu represents citizens and cooperatives to European policy makers, supports the establishment of energy cooperatives, facilitates exchanges between cooperatives, and promotes cooperatives as an alternative business model in the energy sector. It frames itself as empowering citizens in order to achieve energy democracy. As such, it plays a fundamental role in the implementation of the renewable energy transition in Europe.

8 https://eur-lex.europa.eu/legal-content/EN/TXT/PDF/?uri=CELEX:32018L2001&from=EN [accessed on 25 October 2021].

9 https://www.ica.coop/en/cooperatives/cooperative-identity [accessed on 25 October 2021].

10 Indeed, Ostrom’s design principle no. 6 on “Conflict Resolution Mechanisms” states that “Rapid, low cost, local arenas exist for resolving conflicts among users or with officials”, underlining the fact that conflicts are regarded as a burden in common pool resource governance, rather than as something that should be cherished (Ostrom, 2010, p. 653).
